# Ischemic necrosis of the tongue in surgical patients with septic shock: a case report

**DOI:** 10.1186/s12893-016-0164-z

**Published:** 2016-07-19

**Authors:** Jinbeom Cho, Kiyoung Sung, Dosang Lee

**Affiliations:** Department of Surgery, Bucheon St. Mary’s Hospital, The Catholic University of Korea, College of Medicine, Sosa-dong, Wonmi-gu, Bucheon-si, Gyunggi-do, (420-717), Korea

**Keywords:** Tongue necrosis, Tongue ischemia, Septic shock, Ischemic enteritis, Case report

## Abstract

**Background:**

As the tongue is a well-vascularized organ, ischemic necrosis of the tongue is a rare disease entity. Critically ill patients with profound shock may experience end-organ hypoperfusion, which might result in tongue necrosis. However, to our best knowledge, there are no reports regarding ischemic necrosis of the tongue in surgical patients with septic shock.

**Case presentation:**

Two patients recently developed ischemic necrosis of the tongue in our surgical intensive care unit. Both patients had undergone emergent surgery for ischemic enteritis and developed postoperative septic shock. The first patient responded to critical treatment with a short period of circulatory shock, and the delivered dose of the vasopressor seemed to be acceptable. In contrast, the second patient developed postoperative refractory shock, and high-dose vasopressor treatment was required to maintain adequate tissue perfusion. Both patients developed ischemic necrosis of the tongue and died shortly after its emergence, despite vigorous resuscitation.

**Conclusions:**

We suggest that ischemic necrosis of the tongue is an under-reported manifestation of any type of circulatory shock, which may have a complex pathogenic mechanism. Clinicians should be aware of the possibility of ischemic necrosis of the tongue in patients with circulatory shock, even if the patient exhibits clinical improvement, as this awareness may facilitate estimation of their prognosis and preparation for clinical deterioration.

## Background

As the tongue is a well-vascularized organ, ischemic necrosis of the tongue is a rare disease entity, which is generally associated with giant cell arteritis (GCA). Critically ill patients with profound shock may experience end-organ hypoperfusion, which might result in tongue necrosis, although this phenomenon has not been widely reported. One group has reported a series of cases with ischemic necrosis of the tongue among patients with cardiogenic shock [1, 2]; however, to our best knowledge, there are no reports of tongue necrosis in surgical patients with septic shock. We suggest that ischemic necrosis of the tongue can develop as a sequela to any type of circulatory shock, although there has been no evidence to support this hypothesis. Two patients recently developed ischemic necrosis of the tongue in our surgical intensive care unit, and we report these cases to discuss the pathogenesis and clinical implications of tongue necrosis that is associated with shock.

## Case presentation

A comparison of the two cases is presented in Table 1.

**Table 1:** Comparison between the two cases

	Patient 1	Patient 2
Age (years)	88	80
Gender	Male	Female
Clinical presentation	Septic shock	Septic shock
Intraoperative diagnosis	Ischemic necrosis of the colon	Ischemic necrosis of the ileum
Operation	Total colectomy	Segmental resection of the ileum
Possible cause of mesenteric ischemia	unknown	femoral herniation of ileum
Length of vasopressor treatment^a^ (days)	3	until death
Length of mechanical ventilation^a^ (days)	5	until death
Length of CRRT^a^ (days)	until death	until death
Maximum dose of norepinephrine (μg/kg/min)	0.5	2
Maximum dose of epinephrine (μg/kg/min)	0.05	0.5
Coagulation profile^b^		
Antithrombin III (%)	28	13
Fibrinogen (mg/ml)	201	97
FDP (μg/ml)	8.6	18.7
SOFA score at ICU admission^b^	13	16
Respiratory (PaO_2_/FiO_2_)	200	150
Coagulation (Platelet, × 10^3^/μL)	80	40
Liver (Total bilirubin, mg/dL)	1.6	3.4
Dose of norepinephrine (μg/kg/min)	0.5	1
Glasgow Coma Scale score	13	13
Renal system (Creatinine, mg/dL)	2.7	3.6
Blood culture test^b^	Candida species	none
Time to enteral feeding	5th POD	none
Time to necrosis of the tongue	7th POD	8th POD
Pressure on the tongue from endotracheal tube	none	suspicious
Concomitant signs of other organ hypoperfusion	none	Lower limb
Outcome	Died on the 12nd POD	Died on the 10th POD

*CRRT* continuous renal replacement therapy, *FDP* fibrin-degradation product, *SOFA* Sepsis-Related Organ Failure Assessment Score, *ICU* intensive care unit, *POD* post-operative day

^a^includes both the day of operation and the day of complete cessation

^b^checked immediately after the operation

### Patient 1

**Fig. 1:**
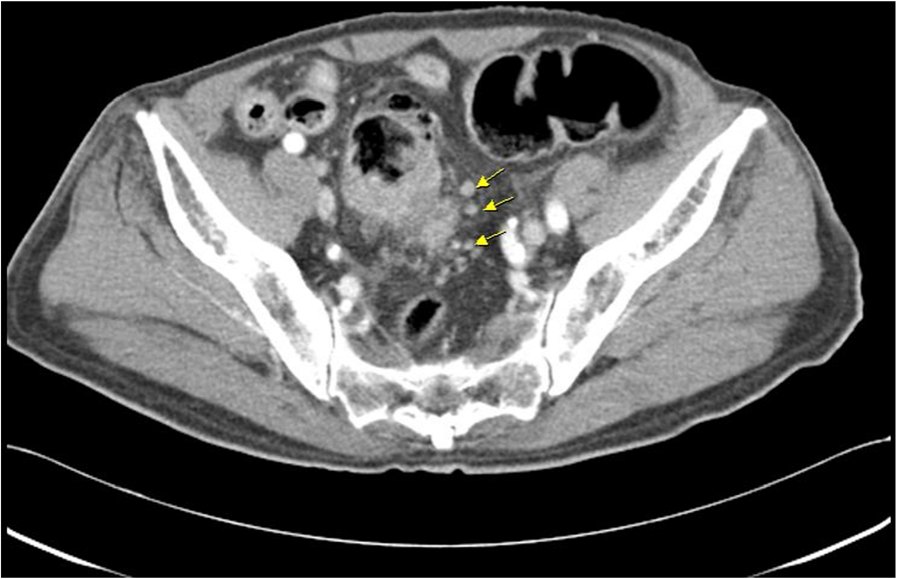
Computed tomography of abdomen shows huge sigmoid colon cancer. Arrows indicate enlarged peri colic lymph node

**Fig. 2:**
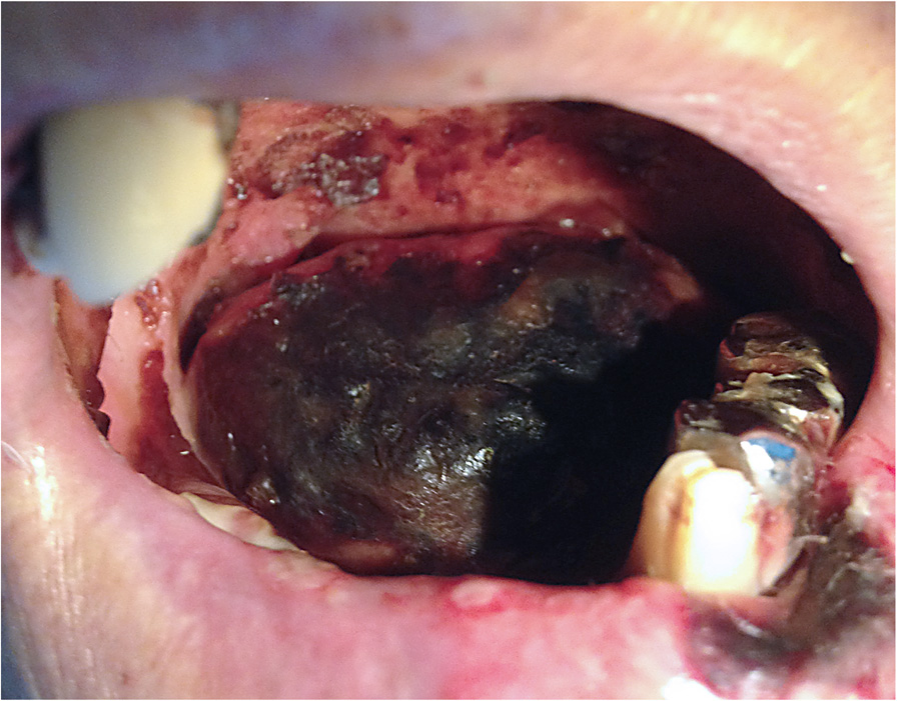
Bilateral gangrene of the oral tongue

An 88-year-old man with hypertension was admitted to the emergency medical center at our hospital based on a complaint of abdominal pain. Abdominal computed tomography (CT) revealed advanced sigmoid colon cancer without any evidence of intestinal perforation or hypoperfusion (Fig. 1). As the patient complained of severe abdominal pain and was hemodynamically unstable, emergent surgery was performed under the suspicion of a surgical emergency. The abdominal cavity was entered through a mid-line incision, and we found ischemic necrosis of the entire colon along with a huge tumor at the sigmoid colon. Therefore, we performed total colectomy with ileorectal anastomosis. There was no evidence of occlusion in the major mesenteric vessels, including the celiac trunk, superior mesenteric artery, and inferior mesenteric artery. After the surgery, we administered critical care for shock reversal and maintenance of adequate organ perfusion. The coagulation profile of Patient 1 suggested disseminated intravascular coagulation (DIC) (Table 1). On the second postoperative day (POD), we ceased the vasopressor and inotropic treatment, and observed that adequate blood pressure was maintained. We weaned the patient off the mechanical ventilation and performed extubation on the fourth POD, and enteral feeding was started via a nasogastric tube on the fifth POD. The patient responded well to the treatment, and we proceeded to the de-escalation phase (with negative fluid balance) of the intensive treatment. However, we noted a darkish coloring of the patient’s tongue on the seventh POD (Fig. 2), and the laboratory findings began to deteriorate (newly noted band neutrophils, decreasing platelet counts, and increasing lactate levels). Because the endotracheal tube had been removed on the fourth POD, it was unlikely that the discoloration was due to the pressure from the endotracheal tube on the tongue. Furthermore, there was no evidence of hypoperfusion in other organs, such as in the limbs or intestines. We performed bed-side duplex ultrasonography and did not detect any occlusive lesions in the external carotid or facial arteries. However, both lingual arteries arising from the external carotid arteries were not visible during the duplex examination. Additionally, bed-side laryngoscopy revealed no mucosal injury in the oral cavity or on the tongue. A meticulous oral hygiene regimen was started, and we administered empirical antiplatelet therapy. However, Patient 1 became hemodynamically unstable and developed multi organ failure on the eighth POD, and subsequently died on the twelfth POD, despite vigorous resuscitation.

### Patient 2

**Fig. 3:**
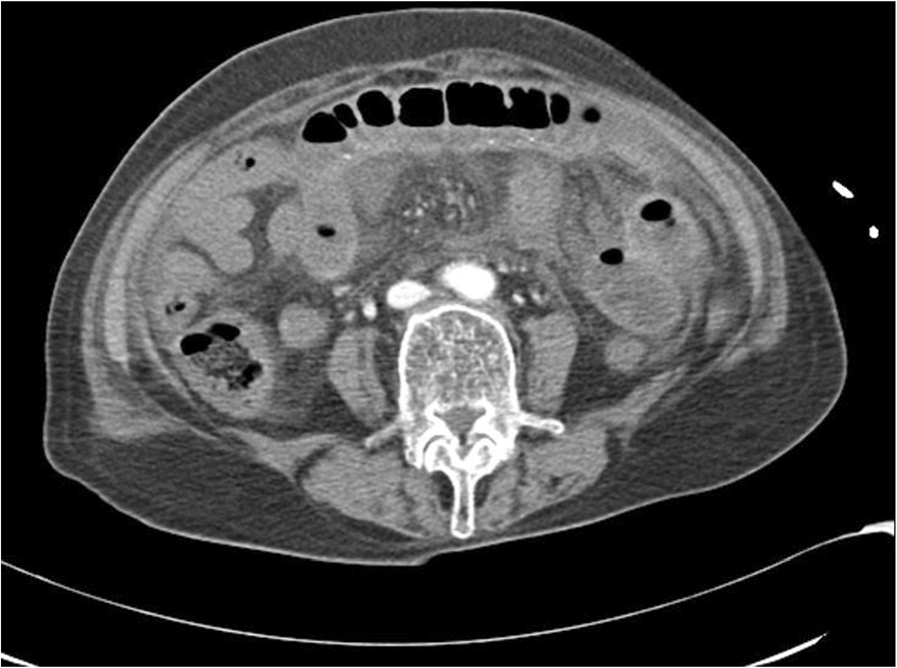
Computed tomography of abdomen shows intraperitoneal free air and decreased perfusion of the small intestine

**Fig. 4:**
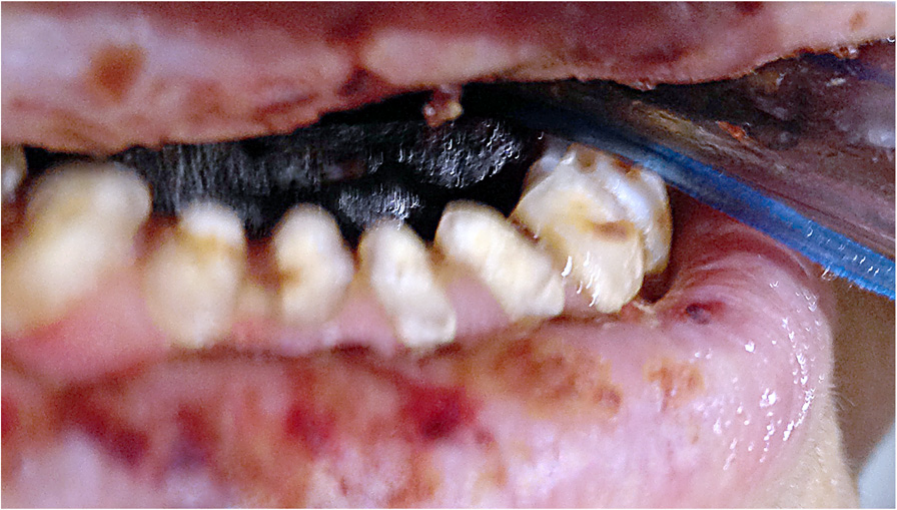
Bilateral gangrene of the oral tongue. The endotracheal tube was placed

An 80-year-old woman underwent herniorraphy for an incarcerated left femoral hernia and there was no evidence of intestinal perforation or hypoperfusion. On the next day, she complained of severe abdominal pain and exhibited profound shock. Abdominal CT scan revealed intraperitoneal free air and decreased perfusion of the small intestine (Fig. 3). Emergent surgery was performed and the findings revealed perforation of the ileum and ischemic necrosis of the small intestine. There was no evidence of major mesenteric vessel occlusion and we resected approximately 20 cm of the necrotic small intestine. After the surgery, the patient was admitted to the surgical intensive care unit for postoperative care, with a clinical presentation of septic shock, severe hypotension, respiratory failure, and anuria. Therefore, we started high-dose vasopressor and inotropic treatment, broad-spectrum antibiotics, fluid resuscitation, mechanical ventilation, and continuous renal replacement therapy to maintain adequate blood pressure and organ perfusion. As shown in Table 1, the coagulation profile of Patient 2 also suggested DIC. We could not taper the intensive treatment because the patient exhibited persistent shock. The high-dose vasopressor treatment (epinephrine at 0.5 μg/kg/min, norepinephrine at 2 μg/kg/min, and no vasopressin) was maintained for 5 days, and tapering was started on the sixth POD. We observed a necrotic change at the tongue on the eighth POD (Fig. 4), as well as critical lower limb ischemia and a worsening in the patient’s general condition. Thus, we performed bed-side laryngoscopy and duplex ultrasonography; however, these examinations did not reveal any diagnostic clues. Unfortunately, we were unable to perform more invasive evaluations (e.g., angiography or biopsy), due to the patient’s poor general condition. We considered tracheostomy, although the patient and her family refused the procedure, and she subsequently died two days later from multi-organ failure. Enteral feeding could not be delivered and mechanical ventilation was performed until the end of the treatment.

## Discussion

Ischemic necrosis of the tongue has occasionally been reported, and is mainly associated with GCA [2]. GCA, or temporal arteritis, is a systemic granulomatous arteritis that involves medium and large arteries, especially branches from the aortic arch, and is mainly found in older women [3]. The cause of this disease is unknown, although it may be an immune-mediated condition [4]. The clinical manifestations of GCA include headache, ocular symptoms, masseteric pain, and tongue pain [3], and systemic corticosteroid therapy is known to be effective for ameliorating the symptoms of GCA. Lingual arteritis can develop in 25 of the patients with GCA, and 15 % of these patients experience lingual artery infarction [4]. In cases of GCA, tongue necrosis may develop secondary to the lingual arteritis, and is typically unilateral, with the exception of one reported case of bilateral tongue necrosis [1, 5]. The diagnosis of GCA usually depends on a clinical suspicion, and arterial biopsy can be performed for unclear cases. The laboratory findings in cases of GCA may include an elevated erythrocyte sedimentation rate, elevated plasma fibrinogen levels, and elevated α-2 globulin levels [4]. Other known causes of tongue necrosis include Kawasaki disease, Wegener’s granulomatosis, DIC, and essential thrombocytosis, although these cases are rarely reported [1].

Tongue necrosis was first reported as a sequela of shock in 2007 [2]. The same group also reported a case series of tongue necrosis that was associated with cardiogenic shock in 2010 [1], and they suggested that impaired perfusion of the tongue in cases of severe shock appeared to be the most reasonable explanation. However, relative blood flow to the heart increases during circulatory shock, and brain perfusion is maintained. Furthermore, the tongue is well supplied by the bilateral lingual arteries and branches of the facial and pharyngeal arteries. Therefore, it is difficult to explain how decreased blood flow to the tongue could occur without concomitant hypoperfusion of the gut, skin, and musculoskeletal system. However, Roman et al. have presented a hypothesis that could explain the hypoperfusion of the tongue during severe shock: blood flow to the internal carotid artery might be protected at the expense of the external carotid system during periods of decreased circulatory volume, and this would reduce the supply of blood to the tongue, which is a potentially susceptible muscular end organ [1].

A search of the literature reveals several other potential causes of tongue necrosis, such as the prolonged use of terlipressin (an anti-diuretic hormone analogue) [6] and DIC [7]. However, it remains unclear what severity of DIC, or what dose of vasopressor treatment, can cause tongue necrosis. As shown in Table 1, the two patients in this report experienced different clinical courses. Patient 1 responded to the critical care with a short period of circulatory shock, and the delivered dose of the vasopressor seemed to be adequate. In contrast, Patient 2 experienced postoperative refractory shock, which necessitated high-dose vasopressor treatment to maintain adequate tissue perfusion. Moreover, Patient 1 did not exhibit any concomitant signs of hypoperfusion to other organs. Therefore, we cautiously suggest that there might be no specific vasopressor dose or severity of shock that must be exceeded to cause ischemic necrosis of the tongue. If the hypothesis that vasopressor or circulatory shock might cause tongue necrosis is correct, other contributing factors are likely needed to induce ischemic necrosis of the tongue during shock. Our cases, and the other reported cases [1], exhibited bilateral tongue necrosis with rapid progression, and the necrosis progressed from the distal section to the proximal section of the tongue, which was less consistent with local pressure from an endotracheal tube and more consistent with a systemic process. This feature was also different from the unilateral necrosis that is observed in cases of GCA, which is a slowly progressing immune disease. Thus, it is possible that no single factor can cause bilateral lingual artery infarction in patients with circulatory shock, and the tongue necrosis may be promoted by complex interactions between the decreased circulatory volume, DIC, vasoconstrictor use, and pressure on the tongue from the endotracheal tube. Therefore, we suggest that tongue necrosis might be induced by any severity of shock, and at any time during the shock treatment, if the interaction(s) between these contributing factors is triggered. Moreover, ischemic necrosis of the tongue might predict a poor prognosis, despite apparent improvements in the other manifestations of shock.

We noticed that our patients developed septic shock secondary to gangrenous necrosis of the intestine. According to the intraoperative findings, Patient 1 seemed to have developed nonocclusive mesenteric ischemia, because we observed no occlusion of the major mesenteric vessels or other causes of ischemic enteritis. This disease entity is considered the end result of the physiological response to a decreased intravascular volume, and the splanchnic vasoconstriction can exhibit a profound and early onset, even before systemic hemodynamic instability arises [8, 9]. Therefore, undetected circulatory compromise in Patient 1 might have proceeded before the gangrenous necrosis of the intestine and septic shock eventually occurred. We hypothesize that the lingual artery infarction might have already developed and that critical end-organ damage at the tongue had progressed, similar to the pathogenesis of the nonocclusive mesenteric ischemia, despite the manifestations of septic shock improving after the operation. However, this hypothesis cannot be applied to Patient 2, because her intestinal necrosis seemed to be caused by mechanical herniation of the intestine.

## Conclusion

In conclusion, we suggest that ischemic necrosis of the tongue is an under-reported manifestation of any type of circulatory shock. The condition may have a complex pathological mechanism, which might consist of interactions between several contributing factors, such as a decreased intravascular volume, DIC, vasopressor treatment, and mechanical pressure from the endotracheal tube. Furthermore, it is possible that no single factor can cause tongue necrosis. Therefore, clinicians should be aware of the possibility of ischemic necrosis of the tongue in patients with circulatory shock, even if they exhibit clinical improvement, as this awareness may facilitate estimation of their prognosis and preparation for clinical deterioration.

## Abbreviations

CT, computed tomography; DIC, disseminated intravascular coagulation; GCA, giant cell arteritis; POD, postoperative day
